# Convergence on Coercion: Functional and Political Pressures as Drivers of Global Childhood Vaccine Mandates

**DOI:** 10.34172/ijhpm.2022.6518

**Published:** 2022-04-05

**Authors:** Katie Attwell, Adam Hannah

**Affiliations:** Political Science and International Relations, School of Social Sciences, University of Western Australia, Perth, WA, Australia.

**Keywords:** Vaccination, Vaccine Hesitancy, Mandatory Vaccination, Policy, Convergence

## Abstract

**Background:** Vaccine hesitancy is a global problem with diverse local policy responses, from voluntaristic to coercive. Between 2015 and 2017, California, Australia, France, and Italy increased the coerciveness of their childhood vaccine regimes. Despite this apparent convergence, there is little evidence of imposition, policy learning, or diffusion – the drivers that are usually discussed in scholarly literature on policy convergence. The fact that the four governments were oriented across the political spectrum, with quite different political and institutional systems, further indicates an empirical puzzle.

**Methods:** To better understand the drivers of enhanced vaccine mandates, a crucial issue during the coronavirus disease 2019 (COVID-19) global rollout, this article engages with four case studies assembled from qualitative analysis of semi-structured in-country interviews and document analysis between November 2018 and November 2020. Key informants had specific expert knowledge or played a role in the introduction or implementation of the new policies. Interview transcripts were coded inductively and deductively, augmented with extensive analysis of legal, policy, academic and media documents.

**Results:** The case analysis identifies two key and interacting elements in government decisions to tighten vaccine mandates: functional and political pressures. Policy-makers in Italy and France were primarily driven by functional challenges, with their vaccination governance systems under threat from reduced population compliance. California and Australia did not face systemic threats to the functioning of their systems, but activists utilised local opportunities to heighten political pressure on decision makers.

**Conclusion:** In four recent cases of high-income jurisdictions making childhood vaccination policies more coercive, vaccine hesitancy alone could not explain why the policies arose in these jurisdictions and not others, while path dependency alone could not explain why some jurisdictions with mandates made them more coercive. Explanation lies in restrictive mandates being attractive for governments, whether they face systemic functional problems in vaccine governance, or political pressures generated by media and activists. Mandates can be framed as targeting whole populations or localised groups of refusers, and implemented without onerous costs or policy complexity.

## Background

**Key Messages**

** Implications for policy makers**

- Vaccination policy does not always respond to objective epidemic risks and is influenced by systemic and public opinion factors.
- Policy-makers can understand the problem factors sitting behind instrument choices in vaccination policy and assess whether they are more functional or more political.
- Policy-makers can understand the drivers towards more mandatory policies (even when they may prefer something more voluntary).
- Policy-makers can assess the importance of other institutions: eg, courts and media, in undermining the functionality or generating political problems within vaccination systems.
- Policy-makers should not be complacent about vaccine uptake (eg, France and Italy) as the problem then becomes harder to deal with.

** Implications for the public**

Vaccination policies may be regarded as responses to objective epidemic risk. However, this research demonstrates that mandatory vaccination policies can arise from a range of drivers because they are attractive for governments facing pressure to respond to lower-than-expected levels of vaccine uptake. For individuals who are wary of coercive policies, this article may serve as a warning to ‘be careful what you wish for.’ Voluntary vaccine uptake needs to be high to weather political pressures towards coercion – and the Australian case shows this may still not be sufficient. Many vaccination experts believe voluntaristic measures are the most appropriate methods for reaching parents of unvaccinated children. However, in the cases examined – Australia, California, France, and Italy – governments responded to pressures by dialling up consequences for those who had not fully vaccinated their children.

Since 2015, governments have expanded or heightened consequences for parents of unvaccinated children^1^ through ‘restrictive’ or ‘hard’ vaccine mandates.^2,3^ In a general sense, governments use mandates to guide population behaviour, imposing consequences to extract compliance from target populations. For example, some countries use taxation to penalise citizens without adequate private health insurance (eg, Australia and the United States until 2019).^4^ With childhood vaccine mandates, consequences include refusal of educational enrolment, fines, and loss of financial entitlements. Historically, jurisdictions with such policies have often permitted exemptions for personal belief (permissive or ‘soft’ mandates, as in Australia and California) or have under-enforced the mandates (as was the case in Italy and France). However, recent reforms in all four jurisdictions have heightened coerciveness, including increasing the number of vaccines covered, imposing new consequences, and removing exemptions.^1,2^

Under-vaccination is a complex problem that states can never fully resolve.^5,6^ It encompasses delay and refusal driven by social, cultural and information ecosystem factors (often called “vaccine hesitancy”), as well as systemic failures to reach (some) populations with free and accessible vaccinations.^7^ Although these problems are attracting considerable public and government attention during the global coronavirus disease 2019 (COVID-19) pandemic, they have well preceded it.

However, vaccine mandates are not the only or necessarily the optimal solution to the factors driving under-vaccination and vaccine refusal. In fact, a strong commitment to persuading publics has prevailed in many industrialised countries (eg, Austria,^8^ the United Kingdom^9^), with targeted persuasive communications^10^ or mandatory informed declinations (ie, counselling or education before an exception is granted)^11,12^ sometimes preferred to coercive policies.^13^ Given the various policy instruments available to governments for improving vaccination rates, the recent convergence on more coercive approaches in Australia, California, Italy and France presents an empirical puzzle. This puzzle is especially of interest given the apparent absence of the usual drivers of convergence identified by scholars of public policy.^14^

Convergence refers to the “growing similarity of policies over time,” and is evaluated by the *degree* of similarity, the *direction *of change (eg, increasing or decreasing regulation) and the *scope* (ie, the number of countries or jurisdictions).^14^ In this paper, we seek to explain why four jurisdictions shifted their childhood vaccine mandates in a more coercive direction at roughly the same time.

Policy scholars have identified several main drivers of convergence. It may be imposed via international agreements or laws; governments may learn from others who have already acted; or policies may diffuse through global networks and epistemic communities.^15^ For example, hospital finance reforms diffused throughout the Organisation for Economic Co-operation and Development in response to rising public health expenditure.^16^

However, there is little evidence of recent convergence on restrictive childhood vaccine mandates resulting from imposition, learning or diffusion. Leading international non-government organisations remain agnostic^17^ or opposed.^18^ Childhood vaccine mandates also lack the support of global vaccination networks^19^ and communities of practice.^20^ Some governments are aware of other jurisdictions’ policies, but at the time of this study there was little evidence of governments systematically analysing or adapting external models for local practice (although such policy importation is now far more common for COVID-19 pandemic vaccinations). Nor was attention paid to long-standing mandatory childhood vaccination policies in former Union of Soviet Socialist Republics satellites and the Global South.^21^ Notably, the jurisdictions studied in this paper changed their policies in such quick succession that there was little opportunity to learn from each other. Despite some coordination between different levels of government *within* some of these countries, the fact that four disparate jurisdictions made their mandatory vaccination policies more coercive than previously, without any apparent coordination *between *them, requires explanation.

One further possibility is that governments experiencing ‘parallel problem pressure’ independently chose similar responses.^14^ This fits the assumption that vaccination policies respond to objective epidemic risk and are adjusted accordingly, with declining vaccine coverage rates or outbreaks of disease provoking a turn to coercion. However, the notion that governments are primarily responsive to objective epidemic risk does not appear to fit the empirical evidence. In fact, diverse local factors appear to have prompted governments to increase the degree of coerciveness of existing mandates^1^ including disease outbreaks^22^ but also court decisions^23^ and community mobilisation.^24^

Might the parallel problem pressure instead be policy-makers’ sensitivity to vaccine hesitancy? Certainly, vaccine hesitancy has been understood to be a global problem for over a decade, and was named as one of the World Health Organization’s (WHO’s) top 10 threats in 2019.^25^ That vaccine hesitancy is an issue in multiple jurisdictions does not, however, explain why it manifests through such different local drivers, nor why in other settings it produces no coercive policy changes at all.^13,26^ The fact that governments introducing restrictive mandates are oriented across the political spectrum, and participate in quite different political and institutional systems (from highly centralised to more regionalised or federalist), further indicates that mandate adoption derives from local pressures. Here, we argue that the missing link lies in the specific kinds of problems that motivate governments to act.

The policy problems governments face have both functional and political dimensions. The functional dimension refers to the degree to which a given policy problem threatens the ongoing and proper function of a broader policy system.^27,28^ Some problems are small in scope or purely technical, requiring recalibration rather than systemic change. They pose more minor functional challenges. In other cases, inaction may risk a potential crisis or spill over into other policy areas. For example, facing rapidly rising expenditure, governments across the world have implemented cost control measures in healthcare^16^ to avoid curtailing health services, and out of concern that budgetary issues would impact other policy areas.^29^

Still, the existence of a functional problem does not *guarantee* action. The rise and fall of policy issues on the political agenda can be explained by the interplay of functional and political elements (Table 1). In other words, the degree to which functional problems create pressure on government to act is shaped by political factors.^30^ Scholars of public policy theorise that governments are motivated by the desire to avoid blame from media, interest groups and, ultimately, voters.^31^ Therefore governments may be incentivised to respond to pressing functional issues because they will be the primary conduit for future political fallout if policy problems have material impacts on citizens.^27^

**Table 1:** Functional and Political Dimensions of Policy Problems

		**Functional **	
		**Low**	**High**
Political	Low	Local, minor, or technical problem; No threat of political losses, simply requires recalibration of existing instruments^35^ For example, readjustment of fee schedules for standard medical procedures^36^	Systemic threat to policy function; no *current *threat of political losses (due to absence of attention or activation of stakeholders and organised interests)^37^ For example, anti-microbial resistance lacks coordinated attention and response^38^
	High	Local, minor, or technical problem, but which still threatens political losses (due to attention or activation of stakeholders and organised interested)^35^ For example, local activism in New Zealand prevents closures of hospitals seen as expendable by successive national governments^39^	Systemic threat to policy function that threatens political losses^27^ For example, lack of health insurance coverage and rising costs create widespread public demands for systemic change in the Unites States.^40^

At the same time, there are always likely to be more policy problems than a political system has the capacity to address. This means that other factors will also be influential in determining which issues provoke government action. As well as their functional dimensions, issues also vary on the degree to which they attract media coverage, activate demands from important constituencies or interest groups, and link to a government’s pre-existing policy agenda.^32^ These factors shape how policy-makers interpret the functional dimensions of policy and can have diverse consequences. In some cases, governments downplay or ignore important policy problems entirely. Equally, it is possible for an issue to attain political attention that is not commensurate with the number of affected people, or the level of threat to a broader policy system.^33^ An example is the Trump Administration’s highly controversial decision to roll back requirements for hospitals and insurers to provide and cover transition-related care for trans Americans.^34^ This change was demanded by conservatives on ideological grounds and impacted trans people’s access to healthcare. However, it was not the result of a government facing the breakdown of a major policy system.

 The enactment of coercive policy solutions such as vaccine mandates may, therefore, serve both functional and political ends. Assessing their interplay helps us to understand why restrictive vaccine mandates have been so attractive for governments despite systemic, partisan, institutional and cultural differences – with important translational lessons for COVID-19 vaccinations.

In tracing the interplay of functional and political problems for childhood vaccination regimes, we address a knowledge gap in existing studies of vaccine mandates. Hitherto, such studies have been brief^1^ or have focused on single countries with little theoretical analysis.^23,41^ One of the few theoretical papers emphasised path dependency as the key explanatory factor for the stability of childhood vaccine governance in Australia, the United Kingdom and the United States.^9^ However, developments in the jurisdictions in this paper require an analytical approach that can explain policy* change*. In paying attention to political factors, we build on earlier studies of pro-vaccine activism^24^ and media contributions to pro-vaccine mobilisation^42-44^ to comparatively analyse the interplay of politics and policy in the tightening of childhood vaccine mandates.

## Methods

Four case studies were assembled from qualitative data collected by the lead author between November 2018 and November 2020 as part of a research project on the introduction, design, and implementation of mandatory vaccination policies in Australia, Italy, France, and California. These cases were selected according to a ‘most different systems’ design,^45^ which compares cases that are similar on the dependent variable but vary in other important respects. All four jurisdictions had recently made their vaccination systems more coercive: expanding the number of vaccines, increasing punishments for non-compliance, or removing opt-outs. Whilst all are wealthy Western democracies, the ‘differences’ include the ideological persuasions of enacting governments, level of governance, health system design, policy history, political culture, and recent epidemiological experiences. California was included as a sub-state unit because, unlike the other jurisdictions, American *states* hold key levers for making childhood vaccination mandatory.^46^ State or regional policy action in Australia and Italy is included within those cases.

Importantly, our application of a ‘most different’ comparative approach does not seek to conclusively evaluate the impact of competing explanatory variables. Instead, we employ ‘theory-guided’ process tracing to elaborate the *mechanisms* by which governments come to decisions, with theory informing the identification of relevant processes and sequences of events to ‘explain the how’ of policy convergence.^47^ For example, while measles outbreaks played a role in three of the four cases, each jurisdiction’s experience was unique in timing, magnitude, and ultimately impact on key policy actors.

 Key informants were identified by the lead author, her in-country advisors, and snowballing. Inclusion criteria consisted of having specific expert knowledge or playing a role in the introduction or implementation of the new mandatory childhood vaccination policy in each jurisdiction. Following ethical approvals, the lead author, in-country advisors, or a previous participant emailed a proposed informant with a brief inquiry about participating in the study. The lead author then sent Participant Information paperwork to the interviewees, who provided formal consent to participate. Interviewees were offered anonymity and approximately half utilised it. Interviewees were asked open-ended questions relevant to their roles, their participation in or knowledge of new vaccine mandates, the processes of agenda-setting, policy design and implementation.

The Italian interviews were conducted in country in November 2018 (5) and September 2019 (3). Two interviews utilised a translator and the rest were in English; one participant was interviewed on both field trips. The Australian interviews (9) were conducted between April 2019 and July 2020. Five were conducted face to face, four via Zoom. The Californian interviews were conducted in June 2019 in country (7) or later that year via Zoom (3) The French interviews (9) were conducted in country in English in September 2020. Supplementary file 1 lists participants in each jurisdiction by role, organisation type and abbreviation. All interviews were transcribed in full by an expert service and checked by the lead author, except the two interviews in Italian, which were transcribed by the translator (a local researcher), machine-translated, then finalised by the translator and checked by the interviewer.

 The lead author analysed all transcripts using NVivo 12 transcription software, employing inductive and deductive coding, with themes generated both by pre-existing research questions as well as emergent findings from the interview data and document studies.

All interviews were augmented with extensive documentary analysis by the lead author, bilingual collaborators, and in-country advisors. This involved cataloguing legal, policy, academic and media documents and, where appropriate, translating them using Deepl machine translation software followed by bilingual checking. Lengthy background analysis was conducted iteratively with the key informant work. Specific information and quotes are directly attributed to some key informants below, but many more informants (as listed in Supplementary file 1) reinforced, explained, and offered nuance to information from media reportage, documents, and published literature. Significant details relevant to elected officials’ adoption of more coercive mandatory vaccination policies in each state are captured comparatively in Table 2.

**Table 2:** Comparative Data Relevant to the Tightening of Vaccination Policies

	**Italy**	**France **	**Australia**	**California**
Year of change	2017	2017 / 2018	2015/2016	2015
DTP vax coverage pre change	93.3% (2016)^48^	99% (2015)^49^	(All vax up to date at 5 years of age) 92% (2014)	(All vax up to date at school entry) 92.8% (2015-2016)^50^
MMR vax coverage pre change	85% (2015)^51^	90.5% (2015)^1^		
Trend of MMR coverage	Declining; rose 2016 ^1,52^	Stable^1^	Increasing^53^	Increasing after 2012 policy^1^
Cases in relevant outbreaks	1620 measles (2017)^54^	>1800 measles (Jan 2017 – Mar 2018^49^	No relevant outbreaks prior	147 measles (2015)
Deaths/hospitalisations	8 (2017-2018) 3263 (2017-2018)^55^	3 (2017)^1^ 23% of cases in one main region (Nouvelle-Aquitaine)^56^	As above	None 20%
Vaccines in mandate^1^	Diphtheria, hepatitis B, Hib, measles, mumps, pertussis, polio, rubella, tetanus, varicella	Diphtheria, hepatitis B, Hib, measles, meningococcal C, mumps, pertussis, pneumococcal, polio, rubella, tetanus	Diphtheria, hepatitis B, Hib, measles, meningococcal C, mumps, pertussis, pneumococcal, polio, rubella, tetanus, varicella	Diphtheria, hepatitis B, measles, mumps, pertussis, polio, rubella, tetanus, varicella

Abbreviations: DTP, diphtheria-pertussis-tetanus; MMR, measles, mumps, and rubella.

## Case Studies

###  Italy

In 2017, the Italian government rebooted its mandatory vaccination regime, first by an executive emergency decree and subsequently by an act of Parliament. A series of compounding events over the preceding decade – court cases, falling vaccination rates, regional governance experiments, and finally a significant measles outbreak – led the government to view existing arrangements as unsustainable.^57^ This was despite many actors investing considerable resources into increasing vaccination rates over several years. Health Ministry officials, who supported elected officials’ eventual resort to stronger mandates, described ‘a lot of movement in different sectors’ with collaborations arising from ‘strong relationships’ with medical and public health experts who produced campaign content, regional actors who organised activities, and a new national vaccination plan. Nevertheless, they ‘weren’t happy’ with the coverage rates from 2016 (IMH2, personal communication).

Italy had historically required four vaccines for children to access school. However, in 1999 the Supreme Court ruled that the right to education took precedent. The remaining sanction of fines was regarded as ineffective and poorly enforced by almost all informants. During this period of unofficial voluntarism, officials believed that Italy would formally adopt voluntary vaccination.^58,59^

Courts would continue to play a problematic role for Italy’s vaccination governance, next chipping away at vaccine confidence.^60^ From 2012, a series of decisions linked vaccinations to autism. A judge in the Trani region led an investigation into this spurious link, generating more bad publicity.^61,62^ These challenges to vaccine confidence were exacerbated by the Fluad scare, in which deaths of elderly Italians were incorrectly linked to receipt of influenza vaccine.^63^ Vaccination rates plummeted, especially the non-mandatory MMR (measles, mumps, and rubella) vaccine, which fell from 90% coverage in 2010 to 85% in 2015.^51^

In 2016, the Emilia Romagna region introduced a new law for the four vaccines that were still ostensibly mandatory, denying unvaccinated children access to early (non-compulsory) education.^62,64^ Regional officials were prompted by falling coverage rates, outbreaks of whooping cough resulting in infant death, a parent advocacy group called “Vaccination in the Nursery,” and supportive local public health bureaucrats (ITE-ER, Venturi, personal communication). Although the new mandate did not include pertussis (whooping cough), this antigen is combined with the mandatory antigens. The Emilia Romagna mandate generated a groundswell of attempts to emulate the policy. Assessore Sergio Venturi – a health official appointed by the region’s elected leader – advocated to the national health minister “that, rather than having fifty different measures, there should be a national one” (personal communication, 2019).

In 2017, a measles outbreak swept through Europe. Its impact in Italy was severe, with 5408 cases.^65^ Officials in the Ministry of Health had grappled with falling rates of childhood vaccination and had been unable to meet misinformation online with their own effective public communications,^57^ with an academic noting “a dramatic lack of organization of communication” (IAC1). Tracing its vaccine coverage rates over several years and familiar with responding to controversies, the Ministry concluded that the population needed clear instruction to overcome widespread ‘no vax’ sentiment in public discourse, identifying that some of the population “need someone else, the institution, who has the authority, saying to them what they have to do,” (IMH2, personal communication). Regional politicians such as Assessore Venturi successfully persuaded Health Minister Lorenzin of the merits of nationalising the Emilia Romagna mandate (ITE1, personal communication). Incorporating previously voluntary vaccines would also incorporate the measles antigen, source of the current outbreak threat (ITE1, personal communication). Compliance would be further encouraged by retaining fines for non-vaccinators.

Italian experts were aware of California’s 2015 policy change^66^ discussed in parliamentary debates (IAC2, personal communication). However, long-term coverage problems, outbreaks, and the sense that existing strategies were inadequate were far more important.^54^ Most Italian public health actors publicly supported the new policy,^67^ which became law in July 2017, and was implemented over the following two years.

###  France

 The French case demonstrates several similarities to Italy: the role of courts, the unhelpful combination of voluntary and mandatory vaccinations, an apparent high rate of hesitancy throughout the population due to scares, amplification of ‘bad press’ for vaccines through the media, and outbreaks of infectious disease. Although these factors manifested differently, the result was similar in generating a perception amongst elected officials and technical experts that the existing governance regime could not continue. This resulted in an act of Parliament to extend France’s vaccine mandate to cover a more comprehensive suite of recommended vaccines. As one technical expert put it, “there was a kind of low but significant, steady decline [in coverage of recommended vaccines] ….and then there were a significant number of people who were loud, in the media, against vaccination” (FTE1, personal communication).

France, like Italy, had long used school and institutional exclusion as a mechanism for making parents accept three older vaccines (polio, diphtheria, and pertussis) for their children, augmented by fines. However, as in Italy, sanctions were rarely applied.^68^ France’s vaccination system had been battered by scares regarding hepatitis B vaccine and H1N1 pandemic vaccines,^24^ during which activists mobilised against vaccines.^69^ Also, as in Italy, mandates did not cover measles. That the vaccination schedule included both mandatory and recommended vaccinations was a problem in both countries, with uptake of the latter lagging by as much as ten percent as parents actively requested vaccines containing fewer antigens.^49^ One technical expert reiterated that “the distinction between mandatory and recommended …was too complicated for most parents” (FTE2, personal communication). This became a much bigger problem when vaccine refusing parents took the French government to court. Their complaint: some ‘voluntary’ vaccines were mandatory in practice – combined antigens meant that parents could not opt-out.

In response to these problems, National Assembly member Sandrine Hurel compiled a report of the policy options.^70^ On her advice, the Health Minister commissioned a citizen’s consultation,^26^ which drew on public opinion and technical expertise to recommend making all vaccinations mandatory.^71^ In 2016, France was found to have some of the highest rates of vaccine hesitancy in Europe^72^; officials in the Ministry of Health and Sante Publique (France’s public health authority) already knew hesitancy was an issue from their own data, as France is one of the few countries that measures public attitudes towards vaccination every four years (FTE3, personal communication). With a measles epidemic in the final quarter of 2017,^49^ the devastation that non-vaccination can cause was clear to decision-makers as they weighed up the policy options. The consultation’s leaders and those in government and public health were also heavily influenced by research suggesting that “if immunisation were not compulsory anymore, the disadvantaged … people would [cease vaccinating],” a finding described by a technical expert as “very important” (FTE4, personal communication). Moreover, citizen feedback indicated concern that public communication about vaccination would be reduced where “the government wants to save money” (FTE4, personal communication), and participants therefore preferred that the government explicitly require vaccination.^23,71^

In early 2017, the Council of State (France’s highest administrative tribunal) issued an ultimatum: change the law or provide a vaccine that separated voluntary and mandatory antigens within 6 months. Private manufacturers were either not willing or able to provide an appropriate vaccine in that timeframe.^23^ But the Council offered one more option – harmonise France’s vaccination policy as either entirely voluntary or entirely mandatory. This cemented the consultation’s recommendations. Incoming Health Minister Agnes Buzyn, a doctor, shepherded through the policy changes formulated under the previous administration (FTE2, personal communication). France imposed an extended mandate on children born from 2018, who would need eleven vaccinations to access care, early education, and school.

###  Australia

The pathway to policy change in Australia was different from the European cases. Australia’s comprehensive mandate already covered all recommended vaccines. In 1998, the national Government introduced the Maternity Immunisation Allowance,^73^ and over subsequent years, children’s vaccination status linked to their parents’ receipt of various cash entitlements and childcare subsidies. A recurrent feature was the Conscientious Objection, rendering Australia’s a ‘permissive’ mandate with capacity for refusal.^2^ Parents were required to vaccinate, obtain a medical exemption, or register as a Conscientious Objector following counselling from a medical provider.^74^

Yet, despite vaccination coverage rates in Australia being high by global standards, including in comparison to Italy and France (especially for MMR), the government was pressured to remove Conscientious Objections. Crucially, and distinct from the European cases, no crisis threatened the ongoing functionality of the system. Rather, actors outside vaccination governance mobilised against vaccine refusal’s *potential* threat. Moreover, rather than the media amplifying vaccine scares and doubts as in Europe, the Australian media was central to the *pro-vaccine* mobilisation.

In 2013, a government report called *Healthy Communities*^75^ released postcode level data showing a small number of regions had coverage rates much lower than the national rate, some as low as 50%. Media reporting focused national attention on this issue, with coverage mobilising vaccinating parents in these regions, who spoke about the risks to their children.^76^ Particular attention was paid to a family who lost their infant daughter to pertussis in 2009, and who immediately received personal attacks from anti-vaccination campaigners. According to the mother, the fact that they “went public…and people saw how we were being treated, that was the catalyst for people to create these [mandatory] policies” (McCaffery, personal communication, 2020).

Meanwhile, in Australia’s largest state, New South Wales, Claire Harvey was investigating day-care centres for her daughter and was “really shocked that you cannot bring peanuts into a day-care centre but you can bring measles, you can bring whooping cough” (personal communication 2019). Harvey used her position as Deputy Editor of the *Sunday Telegraph *tabloid to lobby for removing unvaccinated children from day-care centres and federal subsidies. To galvanise support, Harvey wrote and commissioned over 35 articles and editorials about vaccine refusal, vaccine preventable disease and proposed policy changes.^77^ This included publicising bereaved families’ treatment by anti-vaccination campaigners.^78,79^ Harvey’s “No Jab, No Play” campaign convinced the New South Wales government to require children to be vaccinated for enrolment into early education.^80^

Over the following two years, Harvey targeted national Conscientious Objections through continuing coverage of vaccine refusal. She described members of Australia’s conservative coalition government avoiding her in Parliament because they were lagging with removal of Conscientious Objections, despite having promised to do so (personal communication, 2020), demonstrating that the functional issue itself had failed to capture the attention of policy-makers. Then, in February 2015, The *Sunday Telegraph *reported that the Productivity Commission’s review into childcare arrangements in Australia advocated making rebooted childcare subsidies conditional on vaccination status. In fact, such conditionality already existed, and the report did not explicitly mention Conscientious Objections or removing them.^81^ However, journalist Samantha Maiden characterised the Report as recommending a “tougher line,” quoting both the Social Services Minister and the Opposition Leader as being open to or supporting stronger policy.^82^ The *Telegraph *then mobilised mainstream Australians against “baby-killers”^83^ and alternative lifestyle parents living in “risky hippie hotbeds.”^84^ In April 2015, the *Sunday Telegraph* reported 86% of its readership poll supporting “compulsory vaccination.”^85^

When the national Government finally announced Harvey’s policy, the Prime Minister used her nomenclature of “No Jab, No Pay.”^86^ The new law, passed by the national Parliament in November 2015, shifted Australia to a restrictive mandate by abolishing Conscientious Objections. Eligible and non-medically exempt Australian families would now lose both cash payments and childcare subsidies if they did not vaccinate.^87^

###  California

US states have long required children to be vaccinated against a range of diseases to enrol in school, unless they have a medical exemption. California, like several other US states, also permitted non-medical exemptions (NMEs). American state immunisation laws are regularly contested, with efforts to increase and restrict parents’ capacity to obtain exemptions.^88^ In keeping with the process of many American state legislatures, such changes are often sought by individual members; Californian Assemblyman Dr. Richard Pan successfully led legislative reform in 2012 to make it harder for parents to attain NMEs, requiring counselling from a medical professional.^1^

From late December 2014, a measles outbreak sourced to Disneyland swept through California, affecting 131 locals.^89^ Prior smaller outbreaks had not led to a groundswell of political attention; following an exposure on the Bay Area Rapid Transit system, parent activist Renee DiResta sought action from local politicians regarding personal belief exemptions and met “no momentum for change” (personal communication). In contrast, Disneyland activated many concerned parents like DiResta, as well as political actors and civil society organisations, whose I ♥ Immunity coalition sought to remove personal belief exemptions.^90^ The outbreak played an important role, but in a different way to outbreaks in Italy and France. Europe’s outbreaks provided direct evidence to policy-makers that their systems were under threat, helping to bed down mandate expansions already in motion due to existing problems within the vaccination governance regime. California’s outbreak instead galvanised the community: the abolition of NMEs was at least partly driven from outside the legislature.

As the Disneyland outbreak unfolded, Richard Pan, now a Senator, fielded calls from colleagues and parents seeking legislative action to remove NMEs – the issue was now gathering momentum for change. Pan’s staff connected the parents – whose skillsets including law, policy, public relations, digital merchandising, and social media analysis – who formed the Vaccinate California advocacy group. Pan recalled: “We took the parents who were calling and [who] said “Do something about it!” And we said: OK, we’ll … get them some resources to mobilize” (personal communication). The parents had already been busy – one, Hannah Henry, had secured 20 000 signatures on a petition for change through progressive activism site Moveon.org (personal communication). Importantly, Pan stalled his colleagues until he had the right ingredients for action: parents to act as “the [public] face of the Bill,” and confirmation that the outbreak was now spreading through unvaccinated Californians, so he could demonstrate that “the real problem is that we don’t have enough community immunity to stop the outbreak from going beyond Disneyland” (personal communication). Working with organisations including the California Medical Association, the American Academy of Pediatrics California chapter, the California Immunization Coalition, and the Health Officers Association of California (personal communications with the latter three organisations’ representatives and Pan), the coalition commenced a sophisticated lobbying and public communication effort that garnered wide community and political support for change.^91^

The Shots for School website brought localised data on vaccine refusal to public attention.^92^ This had motivated Hannah Henry, who “[kept] track of the rates over the years” and was “really appalled at … what seemed to be an increase … in unvaccinated children in these kindergartens” (personal communication). Newspapers publicised Shots for School ^93,94^ and the *Los Angeles Times* produced its own searchable database.^95^ Although Californian media did not campaign like the Australian media – instead focusing on more traditional reporting – coverage nevertheless reinforced the coalition’s two key narratives. First, the community lacked sufficient immunity – the Disneyland outbreak being the major evidence – and NMEs were the cause. Second, curtailing NMEs would protect vulnerable members of school communities, including students like Rhett Krawitt, a leukaemia patient,^96^ baby siblings,^97^ and adults with compromised immune systems (DeBurgh, personal communication). Presenting schools as central to broader communities, and current policies as making these communities unsafe, the coalition’s messaging was well-designed to attract publicity and place significant pressure on legislators. By the end of the Bill’s journey through California’s legislature, all but one major state newspaper had come out in support, as had several national papers.^98^

Through skilful mobilisation and framing, as well as sustained lobbying and advocacy, California’s exemption abolitionists won the support of legislative actors. Critically, they convinced the political class to hold the line in the face of unprecedented opposition from vaccine refusing families.^41,99^ Senate Bill 277 was signed into law in July 2015.

## Discussion

 The four cases examined above generate two questions regarding functional and political dimensions of policy problems, which we answer here. First, we consider which pressures led governments to become dissatisfied with the status quo of vaccination governance, including concerns about coverage rates and/or increased hesitancy in the population, and pressures from media and activists. Second, we consider how moving towards the more coercive end of the vaccine governance continuum alleviates these pressures, such as by communicating to the public that vaccines are safe, effective, and necessary, and also by being seen to punish ‘deviant’ refusers. Mandates have additional appeal because they are relatively cheap and easy compared to other forms of intervention for increasing vaccination rates.

###  What Were the Pressures on Vaccine Governance?

 All four cases experienced problems with under-vaccination, which each government came to understand as a behavioural problem. Italian and French officials concluded this from data showing widespread declines in vaccine coverage of non-mandatory vaccinations. In California and Australia, governments were alerted by activists mobilising statistics regarding pockets of refusal amidst otherwise high rates of vaccine coverage.

 However, under-vaccination varied in nature and scope. As a result, while under-vaccination was ‘solved’ by mandates, the pressures it exerted on governments differed between the European cases and the Australia-California cases.

In France and Italy, national problems appeared to threaten the entire machinery of vaccination governance. Widespread non-compliance was compounded by the measles vaccine not being mandatory. System-breaking events occurred within the legal realm, public and social media discourse, and within both jurisdictions’ existing mandatory regimes. Italian regions pursued their own mandates and pushed the national government. France’s Council of State required a new policy in a short space of time, necessitating an ‘all or nothing’ approach. The perception or experience of systemic breakdown was heightened by measles outbreaks in both countries. The *functional* dimension of these problems was of most concern: vaccination regimes might no longer prevent the spread of disease (or in the French case, even be lawful). As such, both governments perceived the need to reboot their existing mandatory regimes to comply with legal requirements (France) and to lift coverage rates, both seeing mandates as communicating the necessity of vaccination to the public.^57,100^

In Australia and California, non-compliance was less severe and more localised. Vaccination coverage rates were generally high, and governments did not regard non-vaccination as a major widespread threat. Instead, community and media activists invoked that prospect to draw media and political attention to low coverage communities. The Disneyland measles outbreak – although significantly smaller than those which would subsequently occur in Europe (see Table 2) – helped Californian activists to demonstrate the salience of this threat. Activists in both settings successfully depicted vaccine refusing families as placing others at risk. In Australia, mobilisation built widespread public support for legislative change, with negative media coverage of vaccine refusers’ behaviour. Californian activists primarily targeted legislators, activating supporters to follow suit. However, both drew government attention to the small group of refusers who enjoyed carte blanche.

###  Why More Coercive Mandates?

Despite the different dynamics, in all four cases, governments moved towards the coercive end of the vaccine governance continuum.^2^ Of course, there is clearly some alignment between the diagnosis of uncooperative individuals – whether localised or widespread – and mandates. However, there are more voluntaristic options for tackling under-vaccination, such as comprehensive and persuasive communication campaigns^57,100^ and invoking descriptive and injunctive norms.^101^ Moreover, while the goal of vaccination is widely shared, debates over the most appropriate policy instruments to achieve it is sometimes polarised on political lines, as in the United States and the United Kingdom,^102^ meaning we should not expect governments to reach for coercive measures to solve any or all vaccine-related problems. Instead, our analysis shows that governments are more likely to reach for mandates where they can quickly – and relatively cheaply – mitigate functional problems and political pressures.

Governments in Italy and France believed their new mandates would strengthen public trust in vaccination as a practice, and in government as the agent encouraging it.^57,100^ In France, this was reinforced by officials’ appraisal of the risks inherent in mandates’ removal. In Italy, regional political factors – such as parental activism in Emilia Romagna – reinforced the functional pressures generated by the country’s declining vaccination rates.

Meanwhile, for governments facing political pressure regarding localised non-vaccination pockets (California and Australia), restrictive mandates met demands for action in a scenario of perceived crisis. In California, measles provided the crisis. In Australia, activists identified “deviant” vaccine refusers whose leadership engaged in unconscionable conduct towards bereaved parents, and whom Australian media presented as a selfish and dangerous ‘Other.’^43,44^ Coercive mandates thus delivered a highly public attack on refusers.

Our cases demonstrate the role of both functional and political factors in leading governments to prefer mandates over more voluntaristic approaches. Buttressing a non-coercive vaccination regime requires multiple policy instruments to cultivate social trust,^103^ a project requiring a much longer timescale and continual inputs. In the context of widespread hesitancy (Italy and France), and with the spread of misinformation through online media, an approach reliant on social trust and public health communication may be fragile.^57^ As noted in our Italian case, government resources may be lacking, and French consultation participants feared that vaccine communications could be impacted by budget austerity. Political will for large-scale public communications may be absent, and was in fact lacking in all our cases.^57,100^ In the context of pockets of refusal (Australia and California), time-consuming and targeted interventions are not as salient as restrictive mandates to a public conditioned by media coverage to think of non-vaccinators as akin to law-breakers (Australia), or to politicians asked to consider refusers’ impact on vulnerable populations (California).

Additionally, restrictive mandates can be implemented with relatively few challenges or costs. They can be rolled out quickly, whether governments perceive a widespread need for behaviour change (Italy and France), or merely that particular groups should no longer be able to opt out (Australia and California). Mandates can increase vaccination without taxing administrative capacity. They push fence-sitters off the fence, helpfully leaving intact the motivations of those who are already vaccinating. Even if mandates prove less effective in communities where refusers cluster,^104^ they can still defuse political pressure by appearing to punish noncompliance. Where mandates already exist – even when not fully enforced – governments can make them more coercive with minimal legislative change. Policies that work largely within existing government capacities have the obvious benefit of being cheaper than those requiring new systems of monitoring, surveillance, and punishment. The Australian government even estimated savings of $508 million over five years by withholding financial benefits from vaccine refusers.^105^ And, as tweaks of existing policies, governments may foreshadow rolling mandates back if future conditions are met, as was promised in both Italy^106^ and France.^71^

###  The Limits of Path Dependence as an Explanation

Because the four jurisdictions already had mandates, ramping up coercion involved relatively simple changes to existing policy instruments. As such, any explanation of these four cases is incomplete without reference to path dependence. However, our analysis also demonstrates the limits of path dependence for describing the mechanisms by which governments choose their policy responses. Path dependence implies structural forces stymieing attempts to break from the existing direction,^107^ yet path dependence is an insufficient explanation for the adoption of more coercive mandates as it offers only limited room for actor agency and for explaining change.^108^ Accordingly, our analysis of functional and political dimensions of policy change has traced the emergence of dissatisfaction with the status quo, highlighting the mechanisms by which existing policy instruments may break down, or be perceived as broken. Bureaucrats, journalists, parents, and politicians embraced more coercive mandates as a solution to hesitancy, confusion, and controversy, or as a punishment for more localised vaccine refusal.

## Conclusion

 In four recent cases of high-income jurisdictions making childhood vaccination policies more coercive, vaccine hesitancy alone could not explain why the policies arose in these jurisdictions and not others, while path dependency alone could not explain why some jurisdictions with mandates made them more coercive. Accordingly, our explanation for new policies in Australia, Italy, France, and California highlights the interaction of functional and political dimensions of policy problems. Mandates can help resolve systemic problems on the one hand, and more local, minor, or technical problems that generate political attention on the other, all without imposing onerous costs or policy complexity on governments.

Although policy learning or diffusion was not a driver of governments’ adoption of mandatory vaccination policies, new policies are already inspiring other jurisdictions. Recent policy changes in New York,^109^ Maine,^110^ and Washington^111^ demonstrate the appeal of the California model as well as activists’ willingness to collaborate across state borders to generate political pressure. Hence, it will remain important to understand the fundamental policy and political conditions that lead governments to view the status quo as untenable.

Additionally, learning and diffusion are not self-generating processes. Governments need impetus to seek out and apply experiences of other jurisdictions, whether from threats to the ongoing function of the vaccination system or pressure emerging from mobilised activists. As such, studies of emerging vaccine mandates will need to integrate understandings of functional and political pressures to understand which lessons are drawn, which knowledge brokers are successful, and why. Given that this present paper only engages with jurisdictions where mandates have been reinforced, it would also be useful to analyse how functional and political pressures have played out in jurisdictions where vaccination policy has *not* become more coercive. Scholars examining these issues in low and middle income countries would additionally need to consider the significant impact of poor state capacity, and whether vaccine distribution and uptake is a key priority of government.

The rollout of COVID-19 vaccines makes our findings even more acute. COVID-19 is a functional crisis *par excellence*: it threatens whole societal domains that would be otherwise taken for granted. As a result, the political environment surrounding the vaccines has become highly polarised. These pressures, combined with the relative ease of introducing coercive mandates, have already led many governments to employ vaccine mandates (also including vaccine passports), seemingly directly importing such strategies from other jurisdictions whilst also drawing on their own pre-existing governance strategies for childhood and health worker vaccinations.^112^ Accordingly, there is an opportunity for analysis of functional and political pressures in vaccine governance on a much larger scale. Local conditions, such as magnitude of cases, decisions relating to access and availability, degree of hesitancy, and broader political polarisation, have varied greatly even as jurisdictions face a broadly similar need for widespread vaccination. Exploring how variation in mandate design and degree of coercion differs between countries and connects to their policy histories as well as functional and political pressures would be fruitful. For example, one could make sense of the Biden Administration’s introduction of a widespread mandate for federal workers, contractors and employees at medical facilities receiving federal funding^113^ based on extremely high case loads and deaths, a flagging rollout, high political polarisation on vaccination, and active obstruction from some states. This could be compared to some Australian states’ heavy use of vaccine mandates,^114^ despite the success of the rollout, since near-successful local elimination created high expectations to keep suffering and death at a minimum. Clearly, the intense pressures governments now face will be important in understanding the choice of tools by which they seek to attain – and maintain – high vaccine uptake.

## Acknowledgements

 The authors thank the many co-researchers, advisors, and research assistants who contributed to the assembling of the case studies. These include Virginia Casigliani, Teresa Garavuzzi, PierLuigi Lopalco, Jeremy Ward, Rebecca Kirkman, Shevaun Drislane, Mark Navin, Julie Leask, Kristine McCartney, and Saad Omer.

## Ethical issues

 Ethics approval for this research was granted by the University of Western Australia Human Research Ethics Committee. Australian interviews were approved by University of Western Australia (UWA) ethics approval RA/4/20/5003 and RA/4/20/5833. Californian interviews were approved by UWA RA/4/20/5326. Italian interviews were authorised by UWA ethics approval RA/4/20/4138. French interviews were authorised by UWA ethics approval RA/4/20/5602.

## Competing interests

 KA receives funding from the Government of Western Australia to conduct research into vaccination attitudes and implementation. The payment goes to the University. KA is an expert advisor to the Australian Technical Advisory Group on Immunisation for COVID-19. This position is unpaid. The committee provides advice to the Australian Government. KA was paid speaker’s fees and travel expenses by Merck/MSD in late 2018 to provide a keynote at an investigator meeting in Poland.

## Authors’ contributions

 Conception and design of the manuscript, acquisition of data, and qualitative analysis was completed by KA. Interpretation of data and drafting of the manuscript was completed by KA and AH.

## Funding

 KA is supported by the Australian Government via the Australian Research Council [Grant Number DE1900015].

## Supplementary files

Supplementary file 1. Table of Interviewees, Jurisdiction and Organisation.
