# Supplementary material for: Convergence on Coercion: Functional and Political Pressures as Drivers of Global Childhood Vaccine Mandates
Source: Int J Health Policy Manag. 2022 Apr 5;11(11):2660–71. doi: 10.34172/ijhpm.2022.6518 (PMC9818102; doi:10.34172/ijhpm.2022.6518)
Supplement: Supplementary file 1 — Table of Interviewees, Jurisdiction and Organisation. [file ijhpm-11-2660-s001.pdf]

Article title: Convergence on Coercion: Functional and Political Pressures as Drivers of Global Childhood Vaccine Mandates

Journal name: International Journal of Health Policy and Management (IJHPM)

Authors' information: Katie Attwell*, Adam Hannah

Political Science and International Relations, School of Social Sciences, University of Western Australia, Perth, WA, Australia.

(*Corresponding author: Email: katie.attwell@uwa.edu.au)

Supplementary file 1. Table of Interviewees, Jurisdiction and Organisation

	**Italy**	**France**	**Australia**	**California**
Public Servants	Ministry of Health (IMH1)<br>Ministry of Health (IMH2)	Ministry of Health (FMH1)<br>Ministry of Health (FMH2)<br>Ministry of Social Cohesion	Chris Baggoley, former Chief Medical Officer, Australia.	California Department of Public Health (CDPH)
Technical Experts	Technical Expert (ITE1)<br>Technical Expert (ITE2)<br>Emilia Romagna (ITE-ER)	Daniel Flores, NITAG.<br>Technical Expert (FTE1<br>Technical Expert (FTE2)<br>Technical Expert (FTE3)<br>Technical Expert (FTE4)	Peter MacIntyre, fmr Director, National Centre for Immunisation Research and Surveillance.	Nicola Klein, researcher, Kaiser Permanente, Northern California
Politicians	Sergio Venturi, Assessore, Emilia Romagna.		Jillian Skinner former NSW Health Minister<br>John Robertson, former NSW Opposition Leader	Richard Pan (Senator)
Academics	IAC 1<br>IAC 2	Didier Torny	AAC 1<br>AAC 2	
Activists / Civil Society			Claire Harvey, *The Sunday Telegraph.*<br><br>Michael Moore, former CEO, Public Health Association, Australia.<br><br>Catherine Hughes, Director, Immunisation Foundation of Australia.<br><br>Toni McCaffery, vaccine advocate.	Vaccinate California:<br>• Leah Russin<br>• Dorit Reiss<br>• Hannah Henry<br>• Renee DiResta<br><br>Kris Calvin, CEO, American Academy of Pediatrics California<br><br>Katherine DeBurgh, CEO, Health Officers' Association California.
